# Properties of stable massive quark stars in holography

**DOI:** 10.1140/epjc/s10052-026-16338-z

**Published:** 2026-09-22

**Authors:** Kazem Bitaghsir Fadafan, Jesús Cruz Rojas, Jonas Mager

**Affiliations:** 1 Faculty of Physics, Shahrood University of Technology, P.O.Box 3619995161, Shahrood, Iran; 2 Departamento de Física, Facultad de Ciencias, Universidad Nacional Autónoma de México, Apartado Postal 70-543, 04510 Mexico City, CDMX Mexico; 3 Institut für Theoretische Physik, Technische Universität Wien, Wiedner Hauptstrasse 8-10, 1040 Vienna, Austria

## Abstract

We study a holographic D3/D7 system, whose dilaton profile has been phenomenologically adjusted in the infrared. The model is used to describe a deconfined yet massive quark phase of QCD at finite density, concluding that the equation of state of such a phase can be stiff enough to support exotic dense stars as massive as 2 solar masses. Nucleons are modeled phenomenologically using the Hebeler et al. EFT baryon phases. For the stiff phenomenological baryon phases the transition to the quark phase is weakly first order allowing for stable quark cores. We also find that holographic baryons, modeled as wrapped D5-branes, provide unrealistic pressures (in the homogeneous approximation) and have to be discarded. We compute the mass vs. radius relation and tidal deformability for these hybrid stars. Contrary to a large number of other holographic models, this holographic model indicates that quark matter could be present at the core of heavy compact stars and may be used to explore the phenomenology of such objects.

## Introduction

Quark stars, a class of compact stars, are believed to form under extreme conditions where the core density is so high that nucleons break down into their constituent quarks, resulting in quark matter [1–4]. A hadron-quark phase transition in the dense cores of neutron stars is considered a plausible mechanism for the formation of quark matter. These stars could emerge from the remnants of supernovae, transitioning from neutron stars if the conditions are sufficiently extreme. Several studies have explored whether quark stars can be formed in nature; in [5] the authors combined information from astrophysical observations and calculations using Effective Field Theory (EFT) and perturbative QCD to argue that in the cores of maximally massive stars, the Equation of State (EoS) is consistent with quark matter. There are also studies using perturbative QCD, which have found that dense quark stars are indeed plausible [6–8].

Recent observations of compact objects through gravitational wave (GW) signals [9] (and their electromagnetic counterparts), mass measurements of the heaviest pulsars [10–14] and mass–radius measurements using X-ray observations by the NICER and other collaborations [15–17], have provided a large amount of data for compact stars. This data places constraints on the dense matter EoS, hinting at the possibility that cores of very massive neutron stars contain a deconfined quark matter phase rather than just a baryonic phase [5]. In this case, the highly compressed nuclear matter phase is expected to undergo a phase transition to deconfined quark matter, releasing its constituent quarks and gluons. Consequently, the EoS of quark matter may be crucial in determining the structure of the star beyond nuclear saturation density. Anticipated advancements in multi-messenger astrophysics offer promising pathways to explore this question [18, 19].

In the astrophysics community one standard way to approximate the EoS is via the MIT bag model [20]. Within the framework of general relativity, one may subsequently explore its impact on the key macroscopic characteristics of compact stars, such as mass and radius. Within the context of modified gravity the static stability criterion, the adiabatic index, and the sound velocity were studied in [21]. However, there are many limitations in such approaches. In the strongly coupled regime there is currently no first principles calculation that can provide the EoS. Despite theoretical hints, quark stars remain hypothetical. Observational challenges, such as their potential resemblance to purely hadronic neutron stars and the uncertainties surrounding the EoS for quark matter, need to be faced to make progress.

First-principles methods, such as lattice QCD, are not well-suited for addressing the regime where quark matter is expected to appear due to the fermion sign problem at large densities. On the other hand, traditional phenomenological approaches such as nucleon EFTs often fail to describe those regimes due to the extreme conditions present in neutron stars where QCD becomes strongly coupled. These problems motivate the exploration of alternative non-perturbative approaches. Holographic models have been used to tackle other challenging problems in QCD, very recently for example in the context of hadronic light-by-light scattering [22–25]. In this work we analyze the predictions of particular models of holographic QCD (hQCD) which originate from phenomenological extensions of D3/D7-brane configurations in AdS/CFT.

hQCD models are inspired by the gauge-gravity duality which relates large $N_c$ strongly coupled QFTs to weakly coupled gravitational theories in higher dimensions. The challenging problem of computing the EoS for a strongly coupled theory like QCD can therefore be approached by working in the dual higher dimensional theory of gravity. Holographic models thus allow one to tackle the strongly coupled regime of matter and predict observable properties of compact stars like the mass–radius relation and cooling behaviors.

Within holography, one can differentiate between top-down and bottom-up models. Top-down models originate from specific, often ten-dimensional, string theory brane constructions where the dual gauge theory is well understood [26–29]. Bottom-up models usually follow the general lessons learned from top down approaches, but there is considerably more freedom in choosing e.g., field content, actions, etc. While the global symmetries and operator content of QCD provide guidance for model building, the bottom-up models lack a precise correspondence to a known field theory. As a result, they contain adjustable parameters that must be tuned to reproduce QCD phenomenology. Compared to top-down approaches the added flexibility is very useful and often necessary to describe important aspects of QCD realistically. In this paper we employ a bottom-up model which is heavily inspired by rigorous D3/D7-brane constructions [27] in IIB string theory.

Among the plethora of bottom-up holographic models, which include the hard- and soft-wall^1^ models [31–33], and models based on Einstein-Maxwell actions as well as more sophisticated models such as the V-QCD model [34], bottom-up models based on the D3/D7 configuration in IIB string theory are especially suitable to describe compact stars [35, 36]. They have enough room for adding ingredients that make the dual theory more realistic, but they are not overly complicated numerically. In this work, we use this kind of model, with a specific non-trivial dilaton profile.

In the holographic D3/D7 setup, the dilaton – a scalar field that determines the coupling in string theory – is chosen to have a phenomenological background profile (while neglecting backreactions to the metric in a first approximation) modeling the running coupling of the dual gauge theory. This modification is also useful in studies of chiral symmetry breaking [37]. Such profiles are designed to interpolate between ultraviolet (UV) and infrared (IR) behaviors, offering a more realistic depiction of the gauge coupling’s energy dependence.

For instance, a dilaton that transitions smoothly from linear in the UV to exponential in the IR can effectively model phenomena such as chiral phase transitions. By tuning its parameters, a wide range of strongly coupled dynamics can be explored. This method draws inspiration from top-down constructions: a well-known type IIB supergravity solution with features of confinement was introduced in [38], while non-supersymmetric backgrounds with deformed AdS geometry and a running dilaton were developed in [39] and applied to chiral symmetry breaking in [40]. These efforts highlight how tailored dilaton profiles help capture essential aspects of gauge theories, such as confinement and chiral symmetry breaking, deepening our understanding of strongly coupled systems. In [35], a restrictive set of dilaton profiles was considered, which additionally had a divergence in the deep IR. The scalar quasinormal modes of this system at finite temperature have been studied in [41]. The behavior of the critical point of QCD-like theories and magnetically induced composite inflation within D3/D7 models have been investigated in [42, 43]. The present work aims to extend analysis of the equations of state to a wider range of profiles for D3/D7 models which are regular in the IR.

Applications of holography to the EoS at nonzero baryon chemical potential and the properties of neutron stars have a rich history [44–48]. Neutron stars and the question whether or not stable quark cores can exist inside of them were investigated in holography within the Sakai-Sugimoto model [49–54], the VQCD-model [55–61] as well as hard- and soft-wall models [62, 63]. In these holographic models quark stars are more or less strongly disfavored.

The study of neutron stars in D3/D7 type models was initiated in [64] in which a constant dilaton, corresponding to the original $\mathcal {N}=4$ SYM setup with probe D7-branes, was utilized (see also [65]). In this model no spontaneous chiral symmetry breaking exists, but a quark mass parameter allows for explicit breaking. The EoS [66] describes a massive deconfined quark phase. It was evident in this first attempt that deconfined conformal matter from holographic models based on the pure D3/D7 case do not yield an EoS which is stiff enough to obtain very massive compact stars.

In [35] the D3/D7 system was modified to incorporate a running anomalous dimension for the quark condensate. This adjustment introduces a dynamic mechanism for chiral symmetry breaking while maintaining a deconfined massive phase at intermediate densities. Interestingly, it was shown that this produces significantly stiffer equations of state and exhibits non-monotonic behavior in the speed of sound, suggesting that such equations of state may be more prone to supporting hybrid stars with quark cores.

Following this important observation, in the present paper we demonstrate that by using a wide range of different effective dilaton profiles which are also regular in the IR (contrary to [35]) one can achieve stable compact stars as massive as 2 solar masses that include quark cores. A scan over the space of parameters reveals a set of allowed EoS that are consistent with the constraints from [67], some of which support quark stars. The improved model also supports stable wrapped D5-branes, which one would in principle identify with nucleons in the dual gauge theory. We show however that (at least in the homogeneous approximation) the resulting nuclear EoS are in conflict with phenomenology and cannot be used in realistic simulations of neutron stars, necessitating the use of the Hebeler et al. EFT EoS [68] for the baryon phase. This model is not able to give a definite answer as to whether stable quark stars exist in nature or not, since that depends on the parameter values chosen. It does however show that such quark stars can *in principle* be obtained from holographic models which allows one to explore the phenomenology of such objects. We find stable quark stars with maximum masses of up to $2.17 M_\odot$ whose tidal deformability drops rapidly upon the formation of quark cores.

The rest of the article is organized as follows. In Sect. 2 we discuss in detail the holographic model we use for the quark matter phase, specifically the dilaton profile responsible for the chiral symmetry breaking mechanism. We also comment on an attempt to include a baryonic phase within the model using D5-branes. In Sect. 3 we show the compact star phenomenology obtained from the model in the previous section. We solve the TOV equations for an ensemble of EoS to find the mass vs. radius curves showing stable massive configurations of quark stars. We also compute the tidal deformability and compare our results to the LIGO/Virgo data. Finally, in Sect. 4 we summarize and conclude.

## Holographic model, quark and baryon phases

In this section we present the model used throughout this work and discuss the different phases it predicts. In Sect. 2.1 we discuss the action, equations of motion and solutions for the quark phase. In Sect. 2.2 holographic baryons as wrapped D5-branes are analyzed. Due to their unrealistic pressures they are discarded in later sections in favor of the phenomenological Hebeler et al. EoS [68].

### Set-up and quark phase

The starting point of the model is the D3/D7-brane configuration [27] dual to the $\mathcal {N}=4$ $SU(N_c)$ SYM with $N_f$ matter hypermultiplets in the fundamental representation, which has $\mathcal {N}=2$ supersymmetry. The dual geometry in the Einstein frame is

1 $$\begin{aligned} ds^2 = {w^2 \over R_{\text {AdS}}^2} (- g_t dt^2 + g_x d\vec {x}^2) + {R_{\text {AdS}}^2 \over w^2} dw_6^2. \end{aligned}$$

The metric functions

$$\begin{aligned} g_t&=\frac{(w^4-w_H^4)^2}{w^4 (w^4+w_H^4)},\\ g_x&=\frac{w^4+w_H^4}{w^4}, \end{aligned}$$

describe a black-hole background with a horizon at $w=w_H$, which encodes a non-zero temperature $T \propto w_H/ R_\textrm{AdS}^2$ in the dual quantum field theory. Although for the purpose of describing cold dense matter, it is sufficient to consider the limit T→0 and thus $w_H\rightarrow 0$, we keep $g_t,g_x \ne 1$ in the analytic formulas up until the numerical implementation in Sect. 3. The AdS radius $R_{\text {AdS}}$ obeys the familiar relation

2 $$\begin{aligned} \frac{R_{\text {AdS}}^4}{\alpha '^2}=4 \pi g_s N_c = \lambda _t, \end{aligned}$$

where $\alpha '=l_s^2$, with $l_s$ the string length, $g_s$ being the string coupling and $\lambda _t$ being the t’Hooft coupling. Considering D7-branes in the D3 background, corresponds to adding $N_f$ quarks to the system. Using for $\mathbb {R}^6$ the coordinate system

3 $$\begin{aligned} dw_6^2=d\rho ^2+\rho ^2 d\Omega _3^2+ d\chi ^2+\chi ^2 d\varphi ^2 \end{aligned}$$

allows one to parametrise the D7-brane embedding by a single function $\chi (\rho )$ . Its world volume can then be described by $(t, \vec {x},\rho , \Omega _3)$ coordinates as long as $\chi (\rho )$ is monotonic. The coordinates χ and ρ are related to *w* via

4 $$\begin{aligned} w=\sqrt{\rho ^2+\chi (\rho )^2}. \end{aligned}$$

The dilaton profile [69]

5 $$\begin{aligned} e^{\phi }=A+1-A ~ \tanh \bigg [\kappa \bigg (\frac{ w-\lambda }{R_{\text {AdS}}}\bigg )\bigg ] \end{aligned}$$

interpolates smoothly and monotonically from 1 in the UV to a finite adjustable value in the IR. The parameters $A,\lambda , \kappa$ determine the precise approach to the IR value. The only scale at zero temperature that breaks the conformal symmetry in the system is λ while *A* controls the IR value of the dilaton, and κ the steepness.

The DBI action for $N_f$ D7-branes in the string frame reads

6 $$\begin{aligned} S_{D7}=-\frac{T_{D7}}{g_s} \int d^{7+1}x e^{-\phi } \text {Tr}\sqrt{-\textrm{det}( G_{\alpha \beta }+2 \pi \alpha ' F_{\alpha \beta })},\nonumber \\ \end{aligned}$$

where $G_{\alpha \beta }$ is the induced (string frame) metric, $F_{\alpha \beta }$ the field strength of a $U(N_f)$ gauge field on the brane and the tension is given by $T_{D7}= (2 \pi )^{-7} \alpha '^{-4}$. The trace refers to the gauge indices. We now switch to the Einstein frame using

7 $$\begin{aligned} G_{\alpha \beta }=e^{\phi /2}G_{\alpha \beta }^E, \end{aligned}$$

where $G_{\alpha \beta }^E$ is obtained by restricting (1) to the D7 brane. The gauge field describes massless degrees of freedom on the D7-brane and encodes the quark chemical potential in its boundary value, as described below. As we are interested in isospin-symmetric nuclear matter, the gauge field can be assumed to be proportional to the identity in flavor space, such that the trace then only supplies an overall factor of $N_f$. Additionally, we assume that the gauge configuration is static and fully described by a single function $A_t=A_t(\rho )$. As shown in [69], this leads to

8 $$\begin{aligned} S_{D7}=-\bar{T}_{D7}~\int ~ dt d^3x d\rho ~\mathcal{L}_{D7}, \end{aligned}$$

where

9 $$\begin{aligned} \mathcal {L}_{D7}= \rho ^3 g_x^{\frac{3}{2}} e^{{\phi }} \sqrt{(1+\chi '^2)g_t- e^{- {\phi }} (\partial _{\rho }A_t)^2 }. \end{aligned}$$

Above we have already transformed to dimensionless $\tilde{\rho }=\rho /R_\textrm{AdS}, \tilde{\chi }=\chi /R_\textrm{AdS}$ using the AdS radius and we have switched to a rescaled gauge field $A \frac{2 \pi \alpha '}{R_{\text {AdS}}} =\tilde{A}$ and subsequently dropped the tildes to simplify the notation. Some of the dilaton factors in (9) arise when expressing the string-frame metric in terms of the Einstein-frame metric with (7). The constant in front of the action reads

10 $$\begin{aligned} \bar{T}_{D7}= T_{D7} \frac{N_f}{g_s} V(S^3) R_{\text {AdS}}^4= \frac{N_f N_c}{(2 \pi )^4}\frac{\lambda _t}{R_{\text {AdS}}^4}. \end{aligned}$$

where $V(S^3)=2\pi ^2$ is the volume of the round 3-sphere. The coordinate $A_t$ is cyclic and its conserved charge is defined by

11 $$\begin{aligned} {d}=\frac{\partial \mathcal{L}_{D7}}{\partial {A}'_t}= \frac{\rho ^3 g_x^{\frac{3}{2}}e^{\phi }\partial _{\rho }A_t}{\sqrt{(1+ \chi '^2)g_t- e^{-\phi } (\partial _{\rho }A_t)^2}}, \end{aligned}$$

which can be used to express $\partial _{\rho } A_t$ in terms of the constant density *d*. Similarly the equations of motion for χ are derived from the Lagrangian (9).

Solving the equations of motion for χ and $A_t$ subject to

12 $$\begin{aligned} \chi (\infty )&= m, \nonumber \\ {A}_t(\infty )&= {\mu }, \end{aligned}$$

one obtains the configurations that are realized in the grand canonical ensemble at fixed chemical potential^2^μ. The parameter *m* describes an analogue of a quark mass in the dual 4d field theory and is set to zero in this paper.

As the equations of motion are second order, one needs one more integration constant for χ and $A_t$ each [40]

13 $$\begin{aligned} \chi (\rho )= &   m+ \frac{c}{\rho ^2}+\cdots \nonumber \\ {A}_t(\rho )= &   {\mu }-\frac{{d}}{2 \rho ^2}+\cdots \end{aligned}$$

The constant *c* measures spontaneous chiral symmetry breaking and *d* is the density of quarks (or baryons after dividing by $N_c$). Their values are determined dynamically by the boundary conditions in the IR. Although later on we exclusively consider the zero-temperature limit $w_H\rightarrow 0$, the topologically different brane embeddings are most effectively explained by turning on a small nonzero temperature.

If $$\sqrt{\rho ^2+\chi ^2(\rho )}> w_H$$ for all $$\rho \ge 0$$ then $\chi^{\prime }\left(0\right)=0$ is necessary together with d=0. If there is a value $$\rho _c$$ for which $$\sqrt{\rho _c^2+\chi ^2(\rho _c)}= w_H$$ then the $$(\rho ,\chi (\rho ))$$ curve in the $$(\rho ,\chi )$$ plane has to intersect $$\sqrt{\rho ^2+\chi ^2}= w_H$$ orthogonally and in addition $$A_t(\rho _c)=0$$. This is because in the Euclidean version of the black hole geometry, the time coordinate is an angular variable and the black hole horizon is just the origin of the coordinate system. The zero temperature case is recovered by letting $$w_H \rightarrow 0$$

**Fig. 1:**
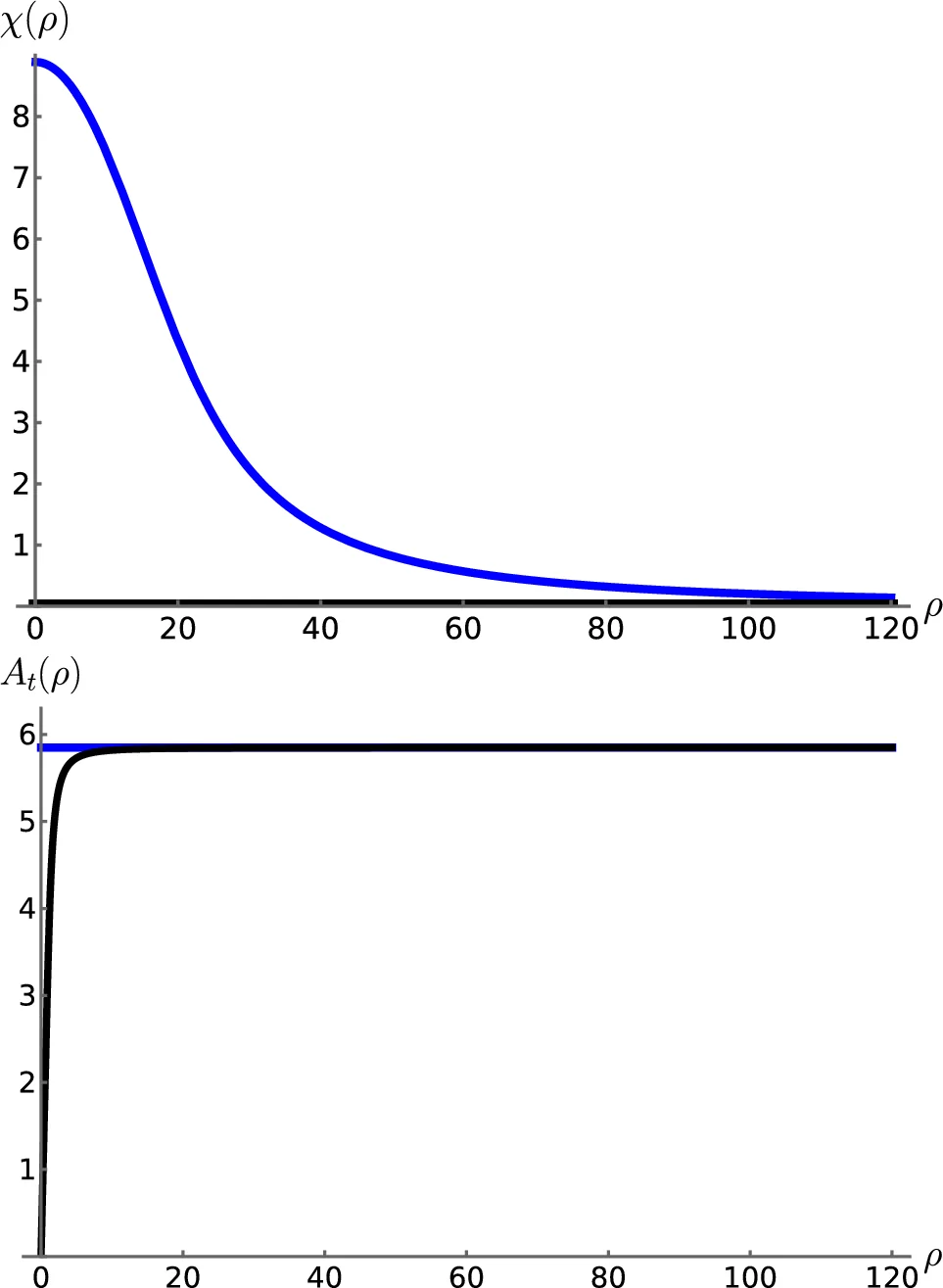
The upper plot shows the embedding function $\chi (\rho )$ in the Minkowski (or vacuum) phase (blue) and the quark phase (black) for a generic value of μ at zero temperature. The lower plot shows the corresponding gauge potentials $A_t(\rho )$. The Minkowski phase has a chiral symmetry breaking vacuum but zero baryon density, while the quark phase restores chiral symmetry and has a nonzero *d*. All quantities are dimensionless and computed for a generic point in parameter space

**Fig. 2:**
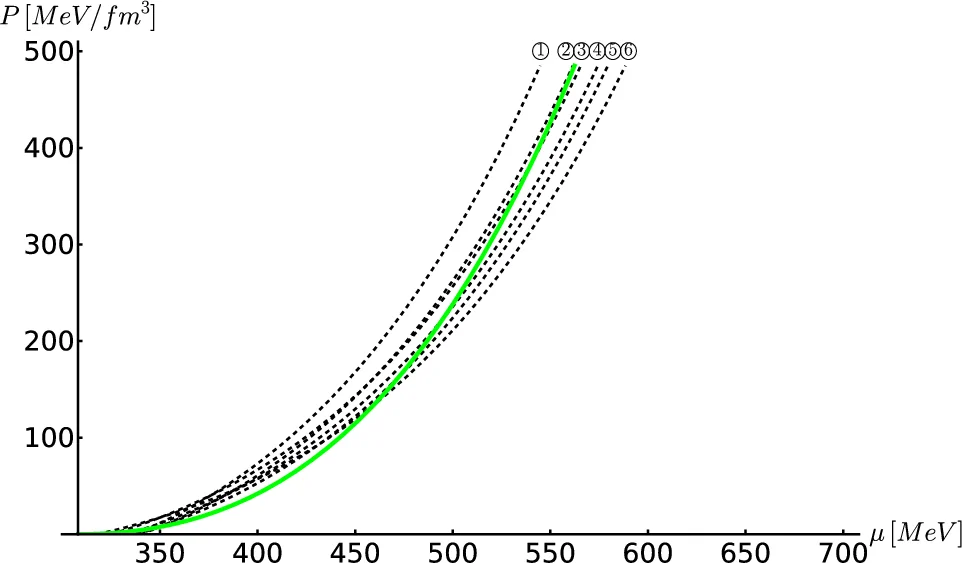
Pressure *P* as a function of the chemical potential for the quark phase defined with dilaton profile (5) and range of parameters $\lambda _t=1.9...3$, $R_{\text {AdS}}= 0.015...0.02$ MeV$^{-1}$ (dashed black). The respective parameters are shown in Table 1. The green curve corresponds to the basic D3/D7 model of [64]. The reduced steepness of some of the the dashed black curves allows for a smoother transition from baryonic matter to quark matter ultimately enabling stars with quark cores when using the stiff Hebeler et al. EoS for nucleon matter

The holographic dictionary equates the on-shell action (stripped off $\int d^3x dt$) $S_{D7}^{os}$ with the grand canonical potential density Ω, which is related to the pressure via

14 $$\begin{aligned} P(\mu )=-\Omega (\mu ). \end{aligned}$$

We call the phase with zero density (for all μ) the Minkowski (or vacuum) phase and its embedding and gauge potential are shown in blue in Fig. 1. Since $$\frac{d}{d \mu } S_{D7}\sim d$$, the action and hence the grand canonical potential density are constant as a variable of μ in this phase. The phase for which $$\sqrt{\rho ^2+\chi ^2}$$ reaches $$ w_H=0$$ is called the quark phase which is depicted in black in Fig. 1. At large μ this phase dominates and at asymptotically large densities reproduces the QCD EoS $$P(\mu )\sim \mu ^4$$.

In Fig. 2 we plot the pressure in the quark phase as a function of the chemical potential for a range of parameters given by $\lambda _t=\{1.9,\ldots , 3\}$, $R_{\text {AdS}}=\{0.015,\ldots ,0.02\}$ MeV$^{-1}$ (we explain our choices for the other parameters in Sect. 3.1) and compare it to the basic D3/D7 model with trivial dilaton of [64] which is also reproduced in Appendix A. The reduced steepness at smaller μ of some of the new curves (with non-trivial dilaton) allows for a smoother transition from baryonic matter to quark matter which leads to stars with quark cores (Table 1).

**Table 1:** Table for the parameter values of the six representative EoS in the figures shown if Fig. 2

EoS	$\lambda _t$	$R_\textrm{AdS}$ [MeV$^{-1}$]
$\textcircled {1}$	1.9	0.015
$\textcircled {2}$	2.4	0.0175
$\textcircled {3}$	2.6	0.0175
$\textcircled {4}$	2.2	0.016
$\textcircled {5}$	3	0.020
$\textcircled {6}$	2.53	0.015

### Baryons as wrapped D5 branes

In this subsection we analyze the baryons intrinsic to the model. As shown in [69] a non-trivial dilaton like (5) can stabilize D5-branes wrapping $S^5$, which have to be interpreted as baryons in the dual gauge theory.

Due to the nonzero background 5-form flux in AdS the world volume gauge field of the D5-brane sees an effective charge $N_c$. The spatial world volume is compact which implies that the total charge on the D5 brane needs to vanish and therefore other charge sources are needed to neutralize this uniform charge density. These are fundamental strings with one end on the D5 brane and one end on the stack of D7-branes. This in turn introduces localized charges on the D7-branes, but here the flux can escape to infinity and contribute to the asymptotic value of (the time component of) the D7 gauge field and hence the density *d*.

To study the D5-brane, we parameterize the $\mathbb {R}^6$ part of the geometry (3) as

15 $$\begin{aligned} dw_6^2 = \textrm{d}\xi ^2 + \xi ^2 \left( \textrm{d}\theta ^2+ \sin {\theta }^2 \textrm{d}\Omega _4^2\right) \,, \end{aligned}$$

The D5-brane will be described by $(t,\theta , \Omega _4)$ coordinates allowing for a non-trivial profile^3^$\xi (\theta )$ where $\theta \in [0,\pi ]$. The brane configurations and relevant coordinate systems in this model are summarized in Table 2.

**Table 2:** This table indicates in which directions and coordinates the various branes in this setup are extended. A dot indicates that the brane stretches in this direction

	AdS-black hole					$S^5$		
Coordinate	*t*	$x^1$	$x^2$	$x^3$	*w*	$S^5$		
Background D3	∙	∙	∙	∙				
					$\mathbb {R}^4$			${\mathbb {R}}^2$
Coordinate	*t*	$x^1$	$x^2$	$x^3$	ρ	$S^3$		${\mathbb {R}}^2$
Flavor D7	∙	∙	∙	∙	∙	∙		
					$\mathbb {R}$	$S^5$		
Coordinate	*t*	$x^1$	$x^2$	$x^3$	ξ	θ	$S^4$	
Baryon vertex D5	∙					∙	∙	

Computing the embedding of a single localized (say near $\vec {x}=\vec {x_0} \in \mathbb {R}^3$) D5-brane, obeying the classical equations of motion, is a very challenging problem. Multi-localized D5 solutions are even more difficult to find.^4^ Only at high densities would one expect to be able to approximate such configurations by a single smeared D5-brane. Localized D5-branes are beyond the scope of this paper and we proceed by using the smeared brane approximation tentatively *for all densities*. As we will show, particularly at low densities there will be some tension between the prediction from the smeared brane approximation and phenomenology, pointing towards the need to localize the D5 branes.

The action of the smeared branes is a sum of DBI and topological terms and is given by

16 $$\begin{aligned} \frac{S_{D5}}{n_{D5}}= &   -\frac{T_{D5}}{g_s} \int {\textrm{d}^{5+1} x ~ e^{-\phi } \sqrt{-\text {det}(\mathcal {G}_{\alpha \beta }+2 \pi \alpha '\mathcal {F}_{\alpha b})}}\nonumber \\  &   +{ 2 \pi \alpha ' T_{D5}\int { \mathcal {F} \wedge C_{(4)}}} \end{aligned}$$

where $\mathcal {G}_{\alpha \beta }$ is the pullback of the string frame metric to the D5 brane and $n_{D5}= \int d^3x \rho _{D5}$ is the total number of D5-branes.^5^ The Chern–Simons term depends on the orientation of the D5 brane, describing either baryons or anti-baryons and, using partial integration can be rewritten as

17 $$\begin{aligned} 2 \pi \alpha ' T_{D5}\int { \mathcal{A}\wedge G_{(5)}}, \end{aligned}$$

where $G_{(5)}=d C_{(4)}$ and $\mathcal {A}$ is the world volume gauge potential so that $\mathcal {F} = d\mathcal {A}$.

The tension of the D5-brane is $T_{D5}= (2\pi )^{-5} \alpha '^{-3}$ and the world volume gauge field of the D5-brane $\mathcal{A}$ (with field strength $\mathcal {F}$) is sourced by the five form $G_{(5)}$ of the background geometry.

The $G_{(5)}$ flux that the background induces is given by

18 $$\begin{aligned} G_{(5)}=2 \kappa _0^2 T_{D3} N_c \frac{d\Omega _5}{V(S^5)}={2}\kappa _0^2 T_{D3} N_c \frac{d\theta (\sin \theta )^4 d\Omega _4}{V(S^5)}, \end{aligned}$$

where $2\kappa _0^2=(2\pi )^7 \alpha '^{4}$, $T_{D3} = {(2\pi )^{-3}} {\alpha '^{-2}}$ and the integration measures on (and volumes of) $S^5, S^4$ are given by

19 $$\begin{aligned} d\Omega _5&=(\sin \theta )^4 d\theta \wedge d\Omega _4, \end{aligned}$$

20 $$\begin{aligned} V(S^5)&=\pi ^3, \end{aligned}$$

21 $$\begin{aligned} V(S^4)&= 8 \pi ^2 /3. \end{aligned}$$

The orientation on the D5-brane is defined such that the worldvolume orientation is $dt \wedge d\theta \wedge d\Omega _4$ (with the standard orientation of $S^4$). This corresponds to a D5-brane (as opposed to an anti-D5-brane). In terms of local coordinates $\theta _1,\theta _2,\theta _3,\bar{\varphi }$ the $S^4$ measure reads

22 $$\begin{aligned} d\Omega _4=\sin ^3 \theta _1 \sin ^2 \theta _2 \sin \theta _3 d \theta _1 d \theta _2 d \theta _3 d \bar{\varphi }. \end{aligned}$$

In (16) the dilaton reads

23 $$\begin{aligned} e^\phi =A+1-A \tanh \left[ \kappa \left( \frac{\xi (\theta )-\lambda }{R_{\text {AdS}}}\right) \right] . \end{aligned}$$

We choose to rescale the dimensionful quantities $\xi = R_{\text {AdS}} \tilde{\xi }$, $\lambda = R_{\text {AdS}} \tilde{\lambda }$ and ${2 \pi \alpha '}\mathcal{A}/{R_{\text {AdS}}} =\tilde{\mathcal{A}}$, where $R_{\text {AdS}}$ is the AdS radius. Through an abuse of notation we will also drop the tilde on $\tilde{\xi },\;\tilde{\mathcal {A}}$ henceforth.

Inserting $\xi =\xi (\theta )$ and $\mathcal{A}_t(\theta )$ into the D5-brane action gives

24 $$\begin{aligned} S_{D5}= &   -\bar{T}_{D5} \rho _{D5} \int dt d\theta \mathcal{L}_{D5}, \end{aligned}$$

with

25 $$\begin{aligned} \mathcal {L}_{D5}&= e^{\frac{{\phi }}{2}} \sin ^4 \theta \sqrt{g_t(\xi ^2{+}\xi '^2){-} e^{-{\phi }}(\partial _{\theta }\mathcal{A}_t)^2 } d\theta \; {-} 4 \mathcal{A}_t \sin ^4 \theta \end{aligned}$$

and

26 $$\begin{aligned} \bar{T}_{D5}=R_\textrm{AdS}^5\frac{T_{D5}V(S^4)}{g_s} \end{aligned}$$

collects all the prefactors except the D5 density, which is explicitly displayed. In the grand canonical ensemble which we consider later, the chemical potential is specified and the D5 density needs to be solved for. It is a dynamical variable just like the embedding function or the gauge field.

The conjugate momentum of ${\mathcal{A}}_t(\theta )$ is defined by $D={ \partial \mathcal{L}_{D5} }/{\partial {\mathcal{A}}_t'(\theta )}$ as

27 $$\begin{aligned} D(\theta )=\frac{\mathcal{A}'_te^{-\frac{{\phi }}{2}}\sin ^4 \theta }{\sqrt{g_t(\xi ^2+\xi '^2)- e^{-{\phi }}(\mathcal{A}_t')^2 }}. \end{aligned}$$

The equation of motion of ${\mathcal{A}_t}(\theta )$ reads

28 $$\begin{aligned} \partial _{\theta } D(\theta )= 4\sin ^4 \theta . \end{aligned}$$

This equation describes the worldvolume gauge fields correctly at all points θ except where the fundamental strings are attached. They would contribute additional delta terms to (28).

The solution of this equation is

29 $$\begin{aligned} D(\theta )= \frac{3 \theta }{2} -\sin ( 2 \theta )+\frac{1}{8} \sin ( 4 \theta ). \end{aligned}$$

**Fig. 3:**
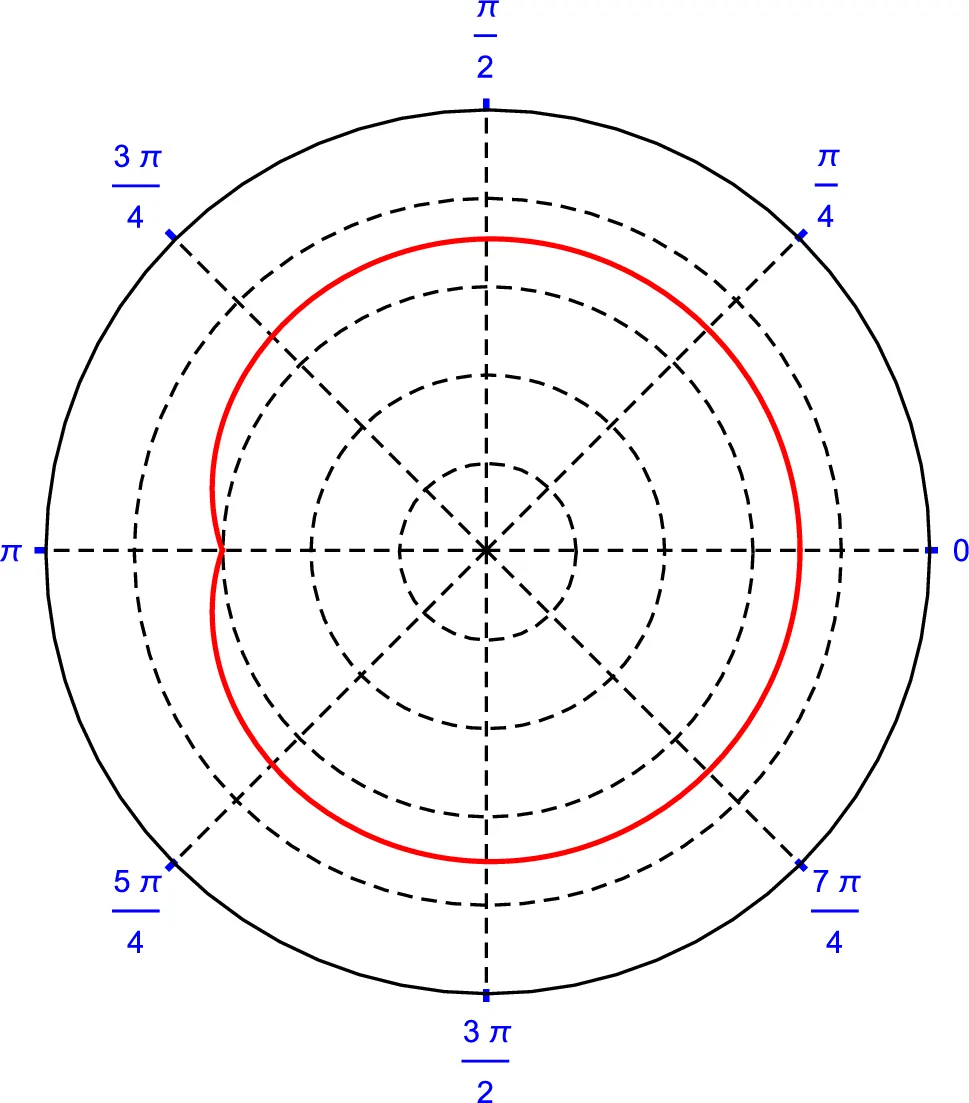
The baryon vertex for $A=10,\tilde{\lambda }=1.715,\kappa =1$. All fundamental strings emerge from the pole at $\theta =\pi$

The integration constant in this expression is zero which implies that the strings emerges only from one of the poles of the baryon vertex which is located at $$\theta =\pi $$. The equation of motion for ξ has to be solved numerically with initial conditions $$\xi (0)=\xi _0, $$ and $\xi^{\prime }\left(0\right)=0$. The non-smoothness of the embedding at $$\theta = \pi $$ is caused by the fundamental strings which attach there, see Fig. 3.

In order to understand the system as a whole we have to also include the D7-branes and the strings connecting the D5 and D7-branes.

The total action density (per unit 3-volume) for the joint system of strings, the smeared D5-brane and the $N_f$ D7-branes reads^6^

30 $$\begin{aligned} -S=&\,\rho _{D5} \overline{T_{D5}} \int d\theta \mathcal {L}_{D5} +\overline{T_{D7}} \int d \rho \mathcal {L}_{D7} \nonumber \\&+\frac{\rho _{D5} N_c R_{\text {AdS}}}{2 \pi \alpha '}\mathcal{A}_t(\theta =\pi )-\frac{\rho _{D5} N_c R_{\text {AdS}}}{2 \pi \alpha '}A_t(\rho =0) \end{aligned}$$

which reduces to the grand potential density when on-shell configurations for the fields are inserted. Energetic arguments suggest that the strings shrink to zero length [70] and hence the area of the fundamental strings vanishes and all that remains are the couplings to the world volume gauge fields in line 2 together with the boundary condition $\xi (\pi )= \chi (0)$.

Every variation of (30) has to vanish at a solution to the equations of motion satisfying the proper boundary conditions. Varying $\xi , \chi$ and the two gauge fields away from ρ=0 and $\theta = \pi$ gives equations of motion already stated above.

Demanding δS=0 for nonzero $\delta \mathcal{A}_t (\pi )$ and $\delta A_t(0)$ results in equations relating the charges of the different branes

31 $$\begin{aligned} \overline{T_{D5}}(2 \pi \alpha ')D(\pi )=N_c R_{\text {AdS}}, \nonumber \\ \overline{T_{D7}}(2 \pi \alpha ')d= \rho _{D5} N_c R_{\text {AdS}}, \end{aligned}$$

Similarly for variations of $\chi , \xi$ that are nonzero at the boundary (but still obey $\delta \xi (\pi )= \delta \chi (0)$) one obtains the force balance condition

32 $$\begin{aligned} \frac{\partial \mathcal {L}_{D7}}{\partial \chi '}\delta \chi |_{\rho =0} =\frac{2}{3 \pi } d \frac{\partial \mathcal {L}_{D5}}{\partial \xi '} \delta \xi |_{\theta = \pi }, \end{aligned}$$

which can be simplified to

33 $$\begin{aligned} \frac{\xi '(\pi )}{\xi (\pi )}= \chi '(0). \end{aligned}$$

Finally as mentioned before, $\rho _{D5}$ is a dynamical quantity and we also need to vary it. The resulting equation is

34 $$\begin{aligned} \frac{2}{3 \pi }\int d\theta \mathcal {L}_{D5}-A_t(0) +\mathcal{A}_t(\pi )=0. \end{aligned}$$

which we solve by setting $\mathcal{A}_t(\pi )=0$ and $A_t(0)= \frac{2}{3 \pi }\int d\theta \mathcal {L}_{D5}$.^7^

The strategy for solving this system of equations numerically is now as follows: for a given value of *d* (or by (31) of $\rho _{D5}$) we solve for $\xi (\pi )$ as a function of the initial value $\xi (0)=\xi _0$. We then use the matching condition $\chi (0)= \xi (\pi )$ and the force balance condition to solve for χ,A as a function of $\xi _0$. This $\xi _0$ is then tuned such that $\chi (\infty )=0$. The (rescaled) chemical potential is then given by

35 $$\begin{aligned} \mu =\frac{2}{3 \pi }\int d\theta \mathcal {L}^{os}_5+\int _0^{\infty } \sqrt{\frac{d^2(1+\chi '^2)g_t}{\rho ^6 g_x^3+d^2e^{-{\phi }}}}. \end{aligned}$$

with on-shell D5 lagrangian

36 $$\begin{aligned} \mathcal {L}^{os}_5=e^{\frac{{\phi }}{2}}\sqrt{(\xi ^2+\xi '^2)g_t}\sqrt{\sin ^8 \theta +D(\theta )^2}. \end{aligned}$$

The on-shell action density is given exclusively by the D7 piece

37 $$\begin{aligned} \Omega =-\overline{T_{D7}}\int d \rho \mathcal {L}_{D7} . \end{aligned}$$

The localized terms in (30) cancel the D5-brane piece upon using (34). Both μ and Ω are thus parametrized by *d* and thus can be used to compute $\Omega (\mu )$. The on-shell action is divergent and still requires holographic renormalisation [71]. The dilaton profile approaches the value 1 in the UV exponentially fast, hence no additional counterterms are required. The renormalization is implemented in practice by integrating only up to a cutoff $\rho _c$, subtracting $\bar{T_{D7}} \frac{\rho _c^4}{4}$ from (37) and taking $\rho _c\rightarrow \infty$.

The relation between the physical chemical quark potential and the above rescaled μ is

38 $$\begin{aligned} \mu = \mu _{\text {phys}} \frac{2 \pi \alpha '}{R_{\text {AdS}}}= \mu _{\text {phys}}\frac{2 \pi R_{\text {AdS}}}{\sqrt{\lambda _t}}. \end{aligned}$$

The rescaled D7-brane tension $\bar{T}_7$ also depends on $R_{\text {AdS}},\lambda _t$ which can be chosen to match with experimental data or with UV QCD. There will be a (dimensionless) value $\mu _{\text {min}}$ of μ such that the grand potential of the D5 phase is the same as that of the Minkowski phase. This point marks the phase transition from the vacuum to a phase with baryons and is in real QCD roughly at $\mu _{\text {phys}}=308$ MeV. Hence we choose $R_{\text {AdS}}=\frac{\sqrt{\lambda _t}}{2 \pi } \frac{\mu _{\text {min}}}{308}$ MeV$^{-1}$. The t’Hooft coupling $\lambda _t$ is a free parameter and can be chosen for example such that the quark phase reproduces the UV QCD result at high chemical potentials.

As mentioned before, the non-trivial shape of the dilaton is a key ingredient in getting stable D5-branes. With a constant dilaton, it is energetically favorable to completely shrink the D5-branes, whilst the profile (5) stabilises them against collapse. As already shown in [69] the resulting phase diagram captures important aspects of QCD qualitatively. The onset phase transition to a baryonic phase at $\mu _{\text {phys}}\sim 308$ MeV which subsequently switches over to a quark phase at higher $\mu _{\text {phys}}$.

**Fig. 4:**
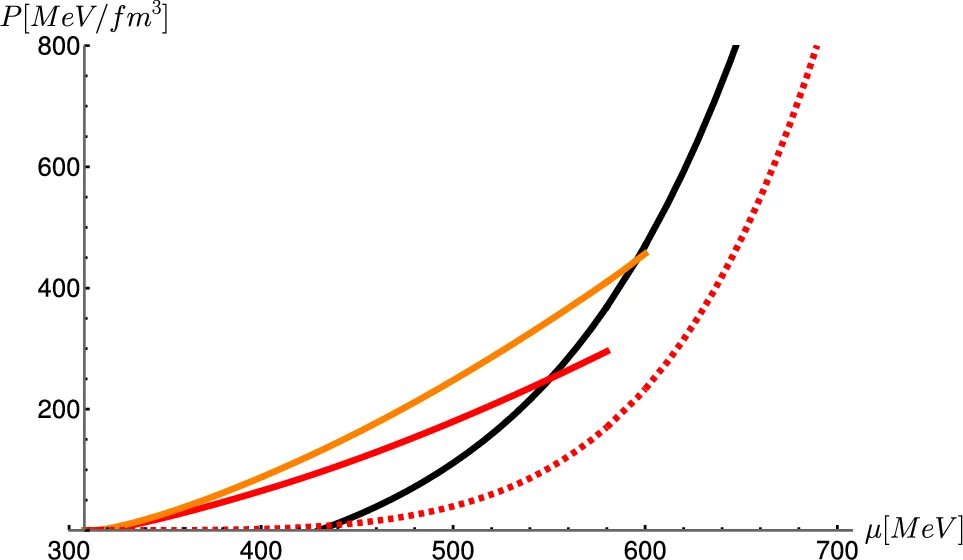
This figure compares the P(μ) behaviors of the *D*5 baryon phase (dashed red) to the phenomenological medium (in orange) and stiff (in red) phases of [68] (we do not consider the soft EoS). The quark phase is shown in black for the parameter choice A=10, λ=1.715, κ=1, $\lambda _t=1.9$

The D5 baryonic phase differs, however appreciably from the phenomenological equations of state of [68] as showcased by the zero-temperature curves in Fig. 4. Particularly just above the transition from vacuum to baryonic matter the D5 EoS compares poorly with the phenomenological equations of state. At small $\mu -\mu _{\text {min}}$ one can use (35) and expand it in the density d to get

39 $$\begin{aligned} \mu \sim \mu _{\text {min}} +d^{\frac{1}{3}} \int _0^{\infty }d \rho \sqrt{\frac{1+\chi '(0)^2}{\rho ^6+ e^{-\tilde{\phi }(0, \chi (0))}}} =: \mu _{\text {min}} +d^{\frac{1}{3}}a, \end{aligned}$$

which holds, provided that $e^{-\tilde{\phi }(0, \chi (0))}$ is non-zero, where *a* is a a density-independent coefficient. Together with $\frac{d}{d \mu }\Omega \sim d(\mu )$ one then obtains $\Omega \sim (\mu -\mu _{\text {min}})^4$ close to $\mu _{\text {min}}$ suggesting a fourth order transition. Equation (39) thus shows that it is impossible to recover a first-order phase transition, as expected of isospin-symmetric QCD, in the present setup for generic dilaton profiles. Similarly, modifications of the action and the force balance condition by various new parameters are also not enough to change the $d^{1/3}$ scaling of the chemical potential near the onset. A non-zero quark mass, implemented by demanding $\chi (\infty )=m$, also results in the same scaling. A potentially more interesting low-density behavior could arise by allowing the strings connecting the D5 and D7 branes to have non-zero extent. Such finite $\lambda _t$ effects would modify the force-balance condition and perhaps also the behavior of the density near the onset. For example in [72, 73] condensation of worldsheet instantons, corresponding to semiclassical strings stretching from the D7 branes to the black hole, strongly impact the order of the phase transition in the D3/D7 model with constant dilaton. It would be interesting to consider such phenomena also for the strings stretching from the D5 branes to the D7 branes.

It may, however, well be that this rather undesirable behavior stems from the smeared approximation and that solutions with localized D5-branes improve the situation at low density. Indeed, within the Witten–Sakai–Sugimoto model it has recently been shown in [74] that a lattice of localized solitonic configurations can be matched to low density nuclear EFT much better than the corresponding homogeneous ansatz. It would be interesting to study such configurations in the present model in the future. The mass of this configuration depends on the parameters of the theory and would provide also an important check of the parameter choice one uses.^8^ Let us also note that localized configurations may also lead to other kinds of phases, such as those found in popcorn transitions [47, 75–77]. In particular in [77] both the the Witten–Sakai–Sugimoto model and the bottom-up V-QCD model are studied and it was found that the popcorn transition appears only in the former model due to the characteristic geometry that ends at the tip of the cigar in the confined phase.

For the moment however our main objective is to describe compact stars that are in agreement with current astrophysical observations, hence in the absence of knowledge about localized D5 solutions we will describe the baryonic phase phenomenologically as in [68]. The parameter $R_{\text {AdS}}$, which was previously fixed to relate $\mu _{\text {min}}$ to $\mu _{\text {phys}}^{\text {trans}}=308$ MeV, can now be varied. In the next section we describe our parameter choices and equations of state. We find a region that, together with the stiff phenomenological EoS, produces stable quark stars whose properties we then explore.

## Quark star phenomenology

In this section we will first describe our choices for the free parameters of the theory and subsequently explore the equations of state and the consequences for neutron star observables such as mass, radius and tidal deformability.

### Parameters

As mentioned before, for the numerical implementation, we, from now on, only consider the $w_H\rightarrow 0$ case. For the parameters $A,\tilde{\lambda }$ we adopt the preferred choice of [69] of $A=10, \tilde{\lambda }=1.715$. There the parameter κ was set to 1 allowing for the stable D5 solutions discussed in the previous section. As we have seen, the resulting nuclear EoS (in the smeared approximation) is rather unphysical and cannot be used as input for neutron star computations. We find that smaller values of κ in general produce weaker first order transitions between baryon and quark matter. We therefore make the choice κ=0.1. We scan over the values of the two remaining parameters $R_{\text {AdS}},\lambda _t$ and highlighting in our plots 6 sample curves. The region $(R_{\text {AdS}},\lambda _t)=(0.015,\ldots , 0.02,1.9,\ldots , 3)$ produces rather stiff EoS with weak first order transitions, some of which weak enough to produce quark stars (those parameter values are mentioned at the end of Sect. 3.3). A more extensive exploration of parameter space is left for future work.

### Equations of state and constraints

**Fig. 5:**
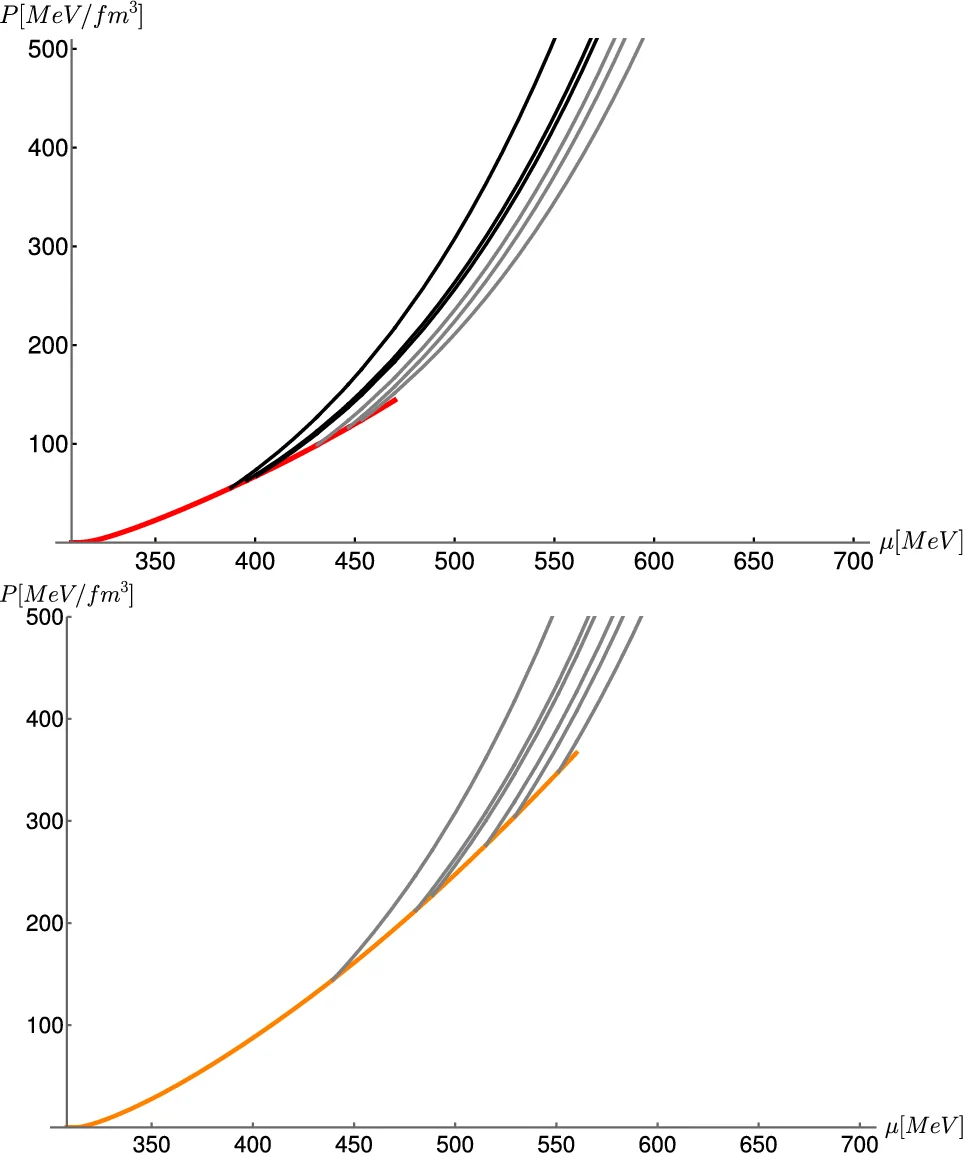
Pressure as a function of chemical potential for the case of stiff (top) and medium (bottom) baryonic matter of [68], and quark matter (black and gray) of the holographic model described in Sect. 2.1. The cases colored in black will support stable quark stars while the ones in gray will not. The parameter choices, which are described in Sect. 3.1, are the same in both plots. The plots only differ in the description of the baryonic phase

With the selection of parameters described in the previous section one can readily compute the pressure as a function of μ and find the transition to baryonic matter. The resulting curves are shown in Fig. 5 for the stiff (red) and medium (orange) Hebeler et al. EoS. In the medium case, the transitions lie mostly around μ=500 MeV, whilst for the stiff EoS the phase transitions happen at considerably lower values of the chemical potential, at around 400 MeV. In the majority of cases (marked in gray), it is not possible to generate stable quark cores, but in the stiff case those with particularly early transitions (marked in black) support quark matter cores as shown in Sect. 3.3.

**Fig. 6:**
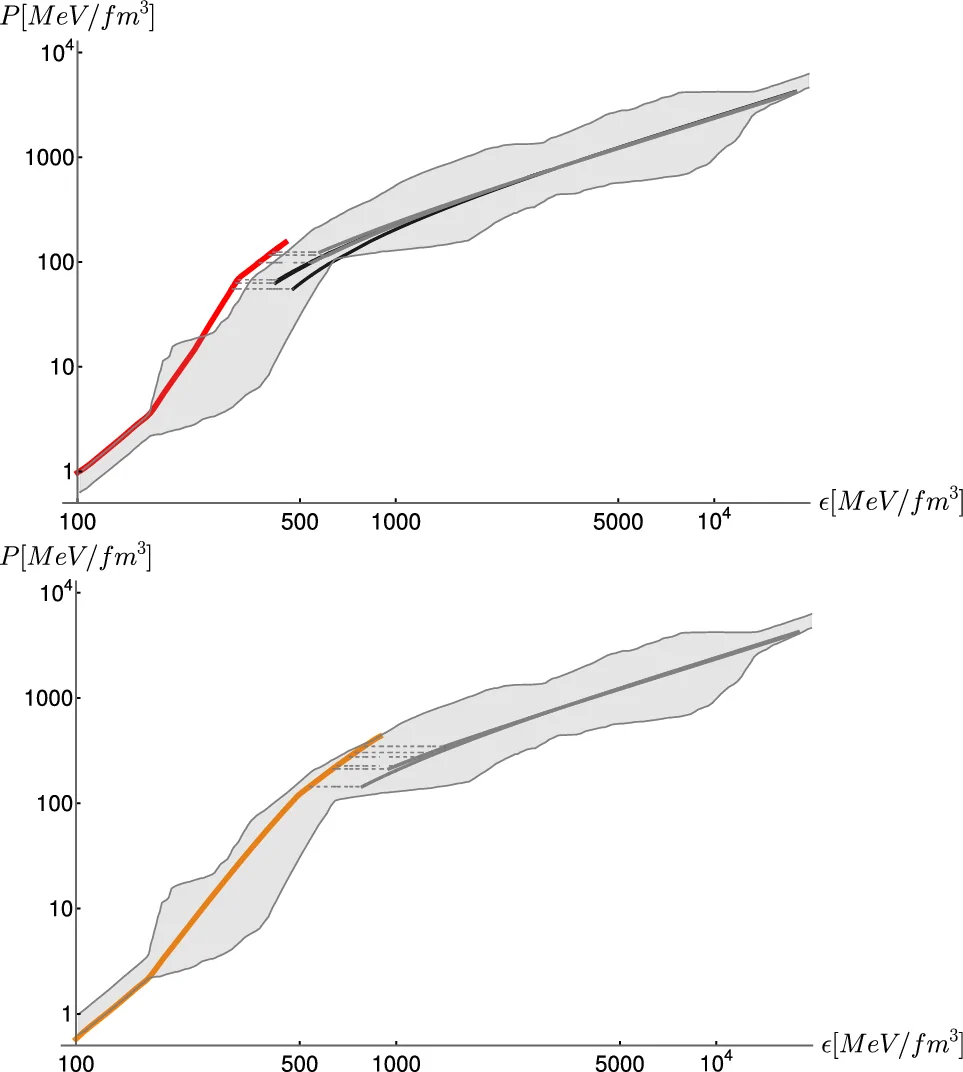
Collection of equations of state in a pressure vs. energy density plot for stiff and medium baryonic phases. Color coding and line styles are as in Fig. 5. Phase transitions are indicated by dashed grey lines. The quark phases all asymptote to the pQCD regime. The gray region found in [67] is consistent with pQCD and astrophysical constraints

One may also study the pressure as a function of energy density ϵ as shown in Fig. 6. The various quark phases all lie within the gray band, which is obtained in [67] by considering a large ensemble of equations of state consistent with astrophysical observations (such as $M_{\text {TOV}}>2 M_{\odot }$ and $\Lambda ({1.4 M_{\odot }})<580$) and pQCD constraints at large ϵ. The stiff baryonic phase is clearly quite close to the upper boundary, and at the locations where the phase transitions happen even slightly outside, of this band. From the point of view of [67] it represents a somewhat extreme EoS, but in our setup it is necessary to have a stiff baryonic EoS to obtain stable quark cores.

**Fig. 7:**
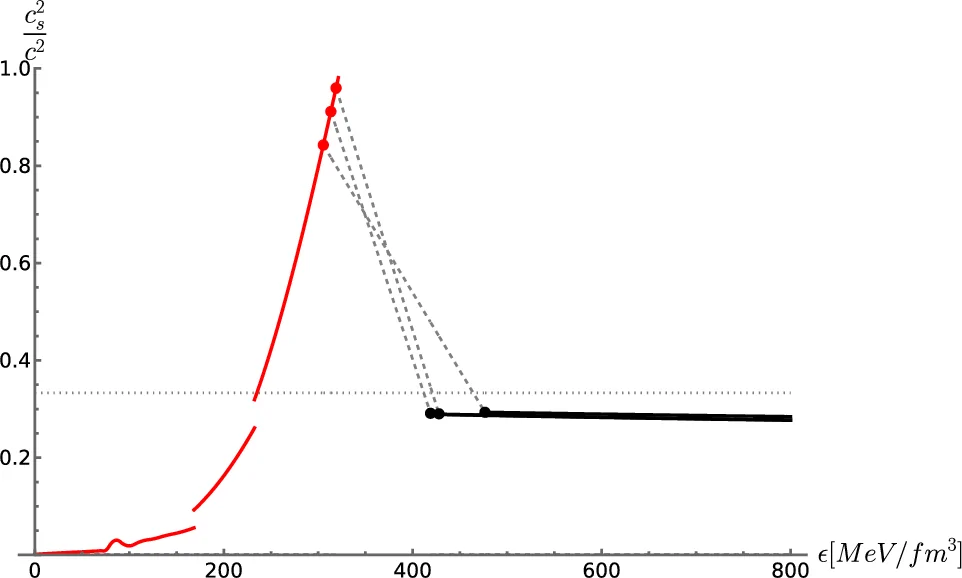
The speed of sound for the 3 equations of state that allow for stable quark matter cores. Dashed gray lines indicate phase transitions to the quark phases, which in this plot lie on top of each other. Although not visible due to the restricted plot range, they approach the conformal $\frac{1}{3}$ (indicated as a vertical dotted gray line) in the UV

The speed of sound for the 3 parameter values which produce quark stars is shown in Fig. 7. The quark phases are barely distinguishable. The speed of sound stays below the speed of light, although the baryonic phase violates the bound from transport $\frac{c_s^2}{c^2}\le 0.781$ found in [78].

**Fig. 8:**
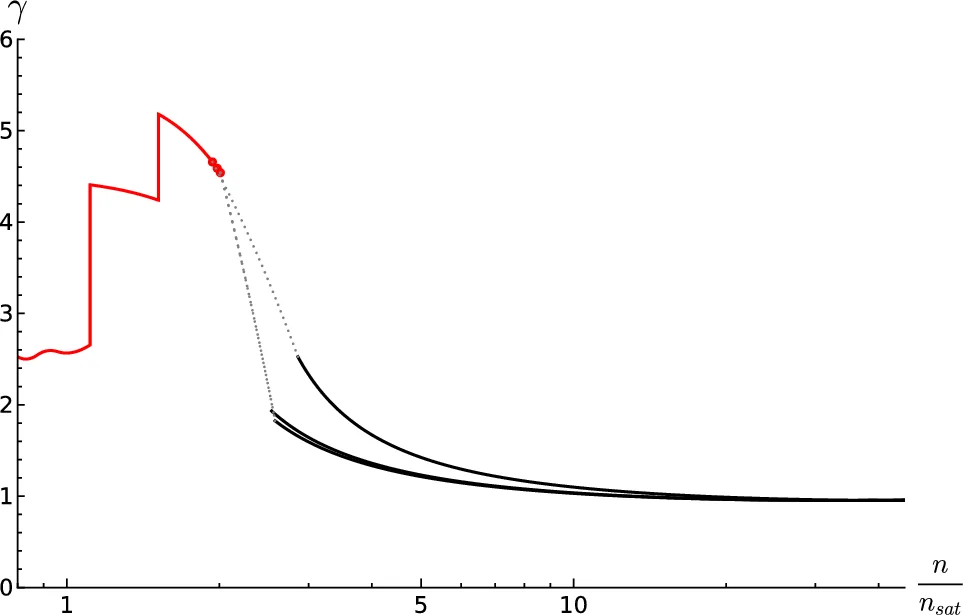
The polytropic index γ for the 3 equations of state that allow for stable quark matter cores as a function of the normalised density $\frac{n}{n_{\text {sat}}}$. Dashed gray lines indicate phase transitions to the quark phases. At large densities, the graphs approach the conformal value of γ=1

For those same 3 equations of state we plot the polytropic index

40 $$\begin{aligned} \gamma (\epsilon )=\epsilon \frac{P'(\epsilon )}{P(\epsilon )} \end{aligned}$$

as a function of the normalized density

41 $$\begin{aligned} \frac{n(\epsilon )}{n_{\text {sat}}}= \frac{1}{n_{\text {sat}}}\frac{\epsilon + P(\epsilon )}{\mu _{\text {phys}}(\epsilon )}, \end{aligned}$$

where $n_{\text {sat}}$ is the nuclear saturation density, in Fig. 8. The polytropic indices in the quark phases clearly exceed γ=1.75, which was used in [79, 80] as a criterion for the onset of quark matter. The results shown in Fig. 8 indicate that this criterion is therefore perhaps too restrictive as in this holographic model quark matter with γ as high as 2.5 can appear. This is to be contrasted with V-QCD studies, which predict transitions at much larger ϵ and a smaller polytropic index γ in the quark phase [58].

### Mass–radius relation

The mass–radius relation for non-rotating compact stars is determined from the EoS by solving the Tolman-Oppenheimer-Volkoff (TOV) equations [81]

42 $$\begin{aligned} \frac{dP}{dr}= &   -G_N\left( \epsilon + P\right) \frac{M+ 4\pi r^3P}{r(r-2G_N M)}, \end{aligned}$$

43 $$\begin{aligned} \frac{dM}{dr}= &   4\pi r^2\epsilon .\qquad \end{aligned}$$

Here *M* and *P* are the mass and pressure in the star as a function of radius *r* and $G_N$ is Newton’s constant. To be able to solve the equations we need the EoS ϵ(P), as well as the central pressure $P_c=P(r = 0)$ as initial condition. The output are the mass *M*(*r*) and pressure *P*(*r*) of the corresponding star. The radius *R* of the star will be the value of *r* at which the pressure vanishes.

**Fig. 9:**
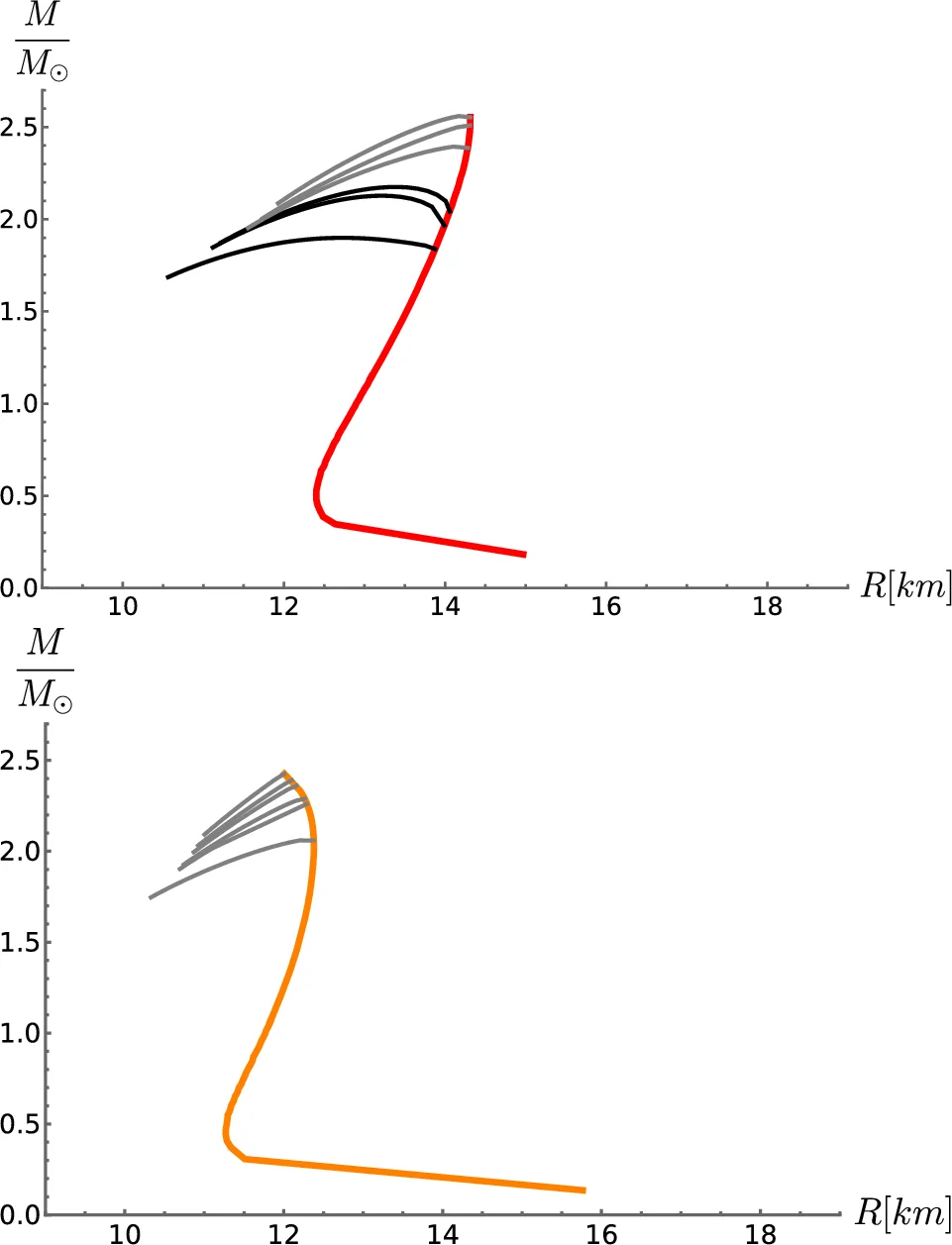
Mass vs radius curves for stiff (top) and medium (bottom) phenomenological baryon phases together with the holographic quark phase. The quark phases highlighted in gray do not lead to stable quark matter in the core, whilst the 3 curves in black in the upper plot support quark stars as high as $M=2.17\, M_\odot$. As mentioned in the text, the stiff baryonic phase is crucial

The results for both the stiff and medium equations of state supplemented by the holographic quark phase are shown in Fig. 9. Initially (at large radii) the stars are fully composed of nucleons. For any fixed parameter choice, quark cores develop when changing from the red (or orange) baryonic curves to the black (or gray) quark curves. A necessary condition for stability is $\frac{\partial M(\epsilon _c)}{\partial \epsilon _c} >0$, where $\epsilon _c$ is the energy density at the center of the star. The stars, whose quark phase is shown in black, obey this criterion until the maximum in the mass vs. radius plot. It is thus possible, with the stiff baryonic EoS, to obtain stable quark stars. See also [82] for a recent analysis of stars with quark cores. When the maximum is passed (from larger to smaller radii), a radial mode becomes unstable and the star will collapse to a black hole. The maximally attainable masses for quark stars in this model for the 3 parameter choices we display are ${M_{\text {max}}}/{M_\odot }=2.17,2.13,1.90$, the first of which is in good agreement (although somewhat favoring lower values) with the currently highest measured mass to date^9^ of $\frac{M^{\text {exp}}_{\text {max}}}{M_\odot }=2.35 \pm 0.17$. From highest to lowest maximal mass, the parameters for the stable quark cores are $(R_{\text {AdS}}\;[\text {MeV}^{-1}],\lambda _t)=(0.0175,2.4),(0.0172,2.33),(0.015,1.9)$.

### Tidal deformability and LIGO constraints

It is expected that, in a binary system of neutron stars during the inspiral process, the tidal forces between the two stars would have measurable effects in the gravitational wave signal that can be observed with gravitational wave detectors. An observable that parametrizes the effect of tidal forces on a neutron star is the tidal deformability $\bar{\lambda }^{(\textrm{tid})}$. It connects the EoS that describes the matter inside neutron stars to the gravitational wave emission during the inspiral. It has been shown that a small tidal signature arises in the inspiral below 400 Hz [83]. This signature amounts to a phase correction which can be described in terms of a single EoS dependent tidal deformability parameter $\bar{\lambda }^{(\textrm{tid})}$, which is the ratio of each star’s induced quadrupole moment due to the tidal field of its companion in the binary system.

We follow references [84–86] to calculate the tidal deformability. In particular it is important to accurately match correctly between the baryonic part of the star and the quark core as first order phase transitions imply special boundary conditions [87, 88]. The dimensionless tidal deformability is defined as

$$\begin{aligned} \Lambda =\frac{\bar{\lambda }^{(\textrm{tid})} c^{10}}{G_N^4\,M^5}. \end{aligned}$$

**Fig. 10:**
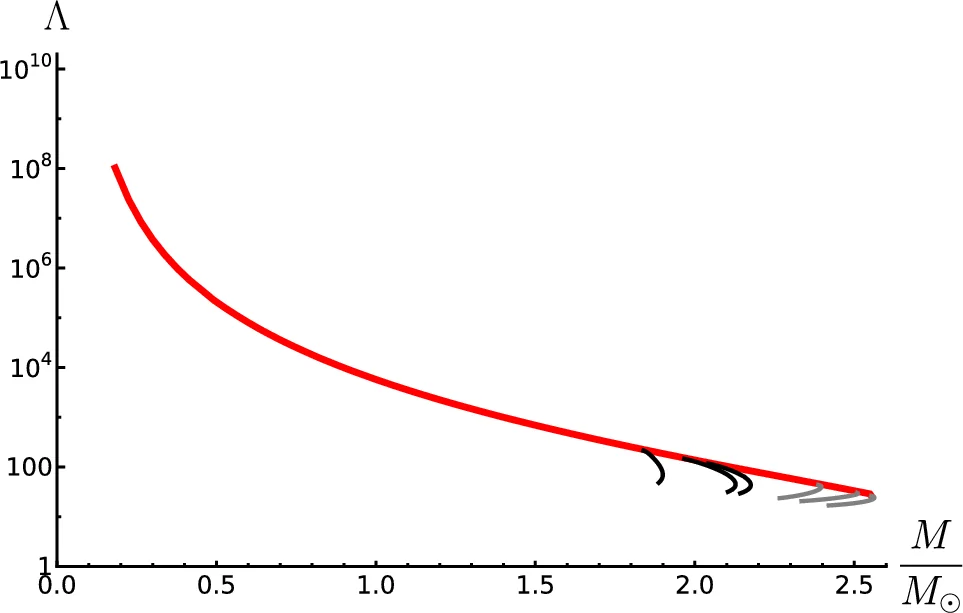
The dimensionless tidal deformability Λ as a function of the mass of the star. After the transition from the phenomenological stiff phase (in red) to the quark matter in the core (in black) the tidal deformability decreases rather rapidly

We plot our results in Fig. 10. In [89] Λ is obtained for the case of effective models of baryonic and strange quark matter. Qualitatively the confined matter curve (red) in Fig. 10 has a similar behavior as the ones obtained from the models in [89]. Once the star gets heavy enough to support quark cores, the tidal deformability decreases rather quickly. It is however difficult to make quantitative predictions as due to the very stiff baryonic phase, the tidal deformability already considerably overshoots the constraint $\Lambda (1.4 M_\odot )=190 ^{+390}_{-120}$ of [90]. The stiff baryonic phase yields $\Lambda (1.4 M_\odot )\sim 950$ and predictions about Λ in the quark core regime are affected by this overestimation in the nucleon phase. Upon formation of quark cores until the maximal allowed mass of the star, the tidal deformability drops by ∼80.

## Conclusions

In this paper we have investigated the D3/D7 model with a modified dilaton profile $e^{\phi }$. The profiles considered interpolate monotonically and smoothly from 1 in the UV to a finite regular value in the IR. Depending on the precise shape of this profile such configurations also allow for baryonic matter in the form of wrapped D5-branes. At least in the smeared approximation the equation of state is however way too stiff and disagrees with phenomenology. Simple modifications of the action or dilaton profile are not able to improve the situation. Due to the absence of a good holographical description of the baryonic phase (at least within this D3/D7 setup) a hybrid approach, utilizing the phenomenological equations of state from [68] and the holographic quark phase, was adopted. These hybrid EoS are largely consistent with existing constraints and for the stiff case are able to produce stable quark stars contrary to most other holographic studies. Furthermore the holographic quark phase produces a polytropic index γ>1.75 for a region of densities just after the transition. The quark matter criterion of [79, 80] is thus violated.

The maximal allowed masses are quite close to experimental data, hinting at the possibility that stable quark cores may exist within detected neutron stars. The tidal deformability shows a quick decrease once quark cores appear. Despite some tensions with existing data, specifically with the tidal deformability constraint [90], this simple model allows one to qualitatively study the implications of a first order transition to quark matter within neutron stars. The stiffest phenomenological EoS leads to some tensions in the tidal deformability and a somewhat large speed of sound. A baryonic EoS in between the stiff and medium phenomenological equations of state fully compatible with existing constraints together with the quark EoS of this paper would likely lead to stable quark cores as well. Interesting observables for future studies include neutrino transport and properties of rotating neutron stars with quark cores.

The phenomenological equations of state used in the computation of neutron star properties however do not follow from any microscopic description. They represent generic, and in the stiffest case, somewhat extreme, states of matter consistent with existing constraints. In order to understand the robustness of our result that quark matter cores may be exist in the heaviest neutron stars, it will be necessary to use a wider range of modern baryonic equations of state or compute it from first principles in a microscopic model.

The properties of neutron stars largely depend on the details of the baryonic phase, hence it would be very interesting to study localized D5-brane embeddings within this model, which presumably more accurately describe the low density regime. Moreover only a rather restrictive region of the parameter space for the quark phase was explored and a more extensive analysis is left for future work.

## Data Availability

This manuscript has no associated data. [Authors’ comment: All data generated or analysed during this study are included in this published article. No separate datasets were generated.]

## Appendix A: Pure D3/D7 quark phase

Let us briefly review the quark phase found in the $\mathcal {N}=4$ duality with $N_f$ D7-branes [27] and worked out in [64]. The DBI action for a probe D7-brane in pure AdS with a constant dilaton is

A1 $$\begin{aligned} S \sim - \int d \rho \rho ^3 \sqrt{1 + (\partial _\rho \chi )^2 - 2 \pi \alpha ' (\partial _\rho A_t)^2} \end{aligned}$$

The brane embedding function $\chi (\rho )$ is holographically dual to the quark mass and condensate and $A_t$ is a gauge field dual to the quark number chemical potential and density. An analytic form for the grand canonical potential density can be found [66]

A2 $$\begin{aligned} \Omega \sim {} -(\mu _{\text {phys}}^2 - m^2)^2 + \mathcal{O}(\mu _{\text {phys}}^3T, T^4)\end{aligned}$$

where *m* and $\mu _{\text {phys}}$ are the asymptotic values of χ and $A_t$ respectively. The string length $l_s$ or $\alpha ^\prime$ (which is formally zero in the supergravity limit) actually cancels from the resulting potential density for the field theory, as usual in the AdS/CFT correspondence.

By suitably fixing $\lambda _t$ one can match the asymptotic pQCD result

A3 $$\begin{aligned} \Omega = - {N_c N_f \over 12 \pi ^2} \mu _{\text {phys}}^4. \end{aligned}$$

The parameter *m* is in principle free and describes quark masses. It has to be bigger than 308 MeV, otherwise the vacuum phase transitions to a quark phase at 308 MeV. This parameter space was explored in [64, 91], where no stable quark stars were found.

## Footnotes

1 Soft-wall models in their most basic form, as well as in a large class of deformations have recently been shown to lead to divergent 4-point functions of QCD flavor currents [30].

2 Note that due to the rescaling of the gauge field the quantity μ is not the physical quark chemical potential.

3 The coordinate ξ is actually the same as the coordinate *w* introduced earlier. Since the D7-brane embedding is already described by a function w(ρ), it is advantageous to nonetheless introduce ξ and describe the D5-brane embedding by the function $\xi (\theta )$.

4 Additionally for any given baryon number, there should be multiple saddle points that contribute. One can always include any given number of baryons-antibaryon pairs without changing the total baryon number.

5 The density $\rho _{D5}$ is related to the density *d* in Eq. (31) below.

6 Both gauge fields have been rescaled and are dimensionless here. The D7-brane coordinates are dimensionless as well.

7 One may add any constant to $\mathcal {A}_t$ without changing the physics. All solutions to (34) are thus equivalent.

8 Although a non-zero quark mass does not lead significantly affect the homogeneous low-density EoS, it may play a more prominent role when considering localized baryons. The quark mass leads to a non-zero pion mass, effectively making the interaction range finite.

9 PSR J0952-0607 has a rotation period of 1.41 ms, whilst our analysis assumed no rotation. Rotating compact objects may exceed bounds valid in the non-rotating case, although for rotation periods of the order of ms only a change of order 1–2% in the maximal mass is expected.

## Code Availability Statement

This manuscript has no associated code/software. [Authors’ comment: The numerical codes are available from the corresponding author upon reasonable request.]
